# Quantitative Analysis and Band Gap Determination for CIGS Absorber Layers Using Surface Techniques

**DOI:** 10.1155/2018/6751964

**Published:** 2018-10-18

**Authors:** Yun Jung Jang, Jihye Lee, Kang-Bong Lee, Donghwan Kim, Yeonhee Lee

**Affiliations:** ^1^Advanced Analysis Center, Korea Institute of Science and Technology, Seoul 02792, Republic of Korea; ^2^Department of Materials Science and Engineering, Korea University, Seoul 02841, Republic of Korea; ^3^Green City Technology Institute, Korea Institute of Science and Technology, Seoul 02792, Republic of Korea

## Abstract

Recently, Cu(In_*X*_Ga_(1−*X*)_)Se_2_ (CIGS) absorber layers have been extensively studied by many research groups for thin-film solar cell technology. CIGS material is particularly promising due to its exceptionally high absorption coefficient and large band gap range, which is adjustable as a function of alloy stoichiometry. To enhance the conversion performance of CIGS solar cells, understanding the CIGS structure and composition is a crucial challenge. We conducted a quantitative study to determine the bulk composition of the major elements such as Cu, In, Ga, and Se of four different CIGS photovoltaic cells. The compositional information was obtained by X-ray fluorescence (XRF), inductively coupled plasma atomic emission spectroscopy (ICP-AES), and femtosecond laser ablation inductively coupled plasma mass spectrometry (*fs*-LA-ICP-MS). Then, the XRF concentration ratio was compared with the intensity ratio of *fs*-LA-ICP-MS to investigate the potential of accurate and rapid analysis using the *fs*-LA-ICP-MS technique. In contrast to the bulk information, the surface techniques can supply detailed information about the chemical composition across the depth profile. Here, elemental depth distributions of CIGS thin films were investigated using magnetic sector secondary ion mass spectrometry (SIMS) and Auger electron spectroscopy (AES). The atomic distributions of four different CIGS absorber layers exhibited a good agreement although they were obtained using two different surface instruments, AES and SIMS. Comparative analysis results of different CIGS absorber layers using SIMS, AES, and *fs*-LA-ICP-MS provide us with the appropriate technique for the information of accurate composition in a rapid analysis time. Thanks to a simple approach using the Ga/(In + Ga) ratio, the optical band gap energy of the Cu(In_*X*_Ga_(1−*X*)_)Se_2_ quaternary layer was monitored in the entire CIGS layer. The elemental distribution and the band gap determination were then used to elucidate their relationship to the corresponding CIGS cell efficiency result.

## 1. Introduction

Cu(In,Ga)Se_2_- (CIGS-)based photovoltaics is one of the most efficient thin-film solar cells due to their unique properties, such as high-absorption coefficient, tunable band gap, and long-term stability. CIGS solar cells have shown notable efficiencies between 21.7 and 22.6% in the laboratory when heavy alkali elements, rubidium and cesium, were incorporated into the substitution of sodium and potassium [1, 2]. Investigating the elemental concentration of CIGS absorber layers is an important challenge to understand the mechanisms that allow CIGS solar cells to exhibit high conversion efficiencies. Indeed, concentration plays a major role because it affects several properties of the CIGS layers, such as morphology, conductivity, carrier concentration, and, more importantly, band gap. For example, an excess of copper concentration in CIGS film induces electrical features such as high conductivity and high doping density from the Cu_2−*X*_Se secondary phase, which is not a single-phase solar cell, but plays a key role in grain growth [3]. The morphology varies with the copper concentration in the CIGS thin film, while the phase transformation depends on the substrate temperature [4, 5]. In addition, the Ga/(In + Ga) ratio is known to affect the band gap of absorber layers, which can be tuned by the Ga content [6–8]. CIGS thin films deposited by a three-step process increase the Ga composition toward the Mo layer, which causes an increase in the band energy. An increased band gap close to the Mo back contact reduces the recombination at the CIGS/Mo interface by accelerating the generated electrons to the PN-junction [9, 10]. Therefore, analysis of the composition of the absorber layers and characterization of the compositional distribution as a function of depth are essential.

Many research groups have studied the composition of CIGS absorber layers through inductively coupled plasma atomic emission spectroscopy (ICP-AES), X-ray fluorescence (XRF), and wavelength-dispersed electron probe microanalyzer (EPMA) [11–14]. Recently, femtosecond laser ablation inductively coupled plasma mass spectrometry (*fs*-LA-ICP-MS) has been applied for the analysis of CIGS solar-cell film due to the sensitivity, rapid analysis, and minimal consumption of the region for an elemental analysis [15–18]. However, the above techniques have limitations in measuring the elemental concentration as a function of depth, and thus surface analytical techniques such as secondary ion mass spectrometry (SIMS) and Auger electron spectroscopy (AES) have been widely used [19–23]. The SIMS technique, with its high detection limit and ability to obtain the profiles of elements in parallel, is a powerful tool for investigating trace and constitutional elements in CIGS absorber thin films.

Here, four different CIGS absorber layers were fabricated by a three-step coevaporation process. The average compositions of the major elements in the CIGS absorber layers were measured by ICP-AES and XRF measurements. *fs*-LA-ICP-MS was also used for elemental analysis of the CIGS thin films, with the advantages of rapid analysis, minimal sample consumption, and no sample preparation. Surface techniques were compared with other analytical methods for the quantitative determination of the composition of the CIGS thin films. The bulk compositional techniques such as XRF, ICP-AES, and *fs*-LA-ICP-MS lead to various advantages for the analysis of the CIGS thin films. They are sensitive, accurate, and rapid methods without vacuum system so that they can be used complementarily to the surface techniques for the CIGS analysis. Depth profiles from SIMS and AES were used to compare the compositional distributions and to determine the band gap in the CIGS solar cells.

## 2. Materials and Methods

### 2.1. CIGS Solar-Cell Preparation

The Cu(In,Ga)Se_2_ solar cells were produced at the solar-cell center of the Korea Institute of Science and Technology (KIST). Four different CIGS solar cells were fabricated with a similar stack structure, i.e., Al-doped ZnO (250 nm)/i-ZnO (50 nm)/CdS (50 nm)/CIGS (2 *μ*m)/Mo (500 nm) on a soda-lime glass (SLG) substrate. The bilayer Mo film, consisting of a 100 nm thick porous bottom and a 400 nm thick dense top, was deposited by direct current sputtering. The CIGS absorbers were prepared with four different conditions to clarify the influence of the experimental conditions on the cell efficiency. Three absorber layers, B01, B02, and B03, were fabricated on a bilayer Mo film on an SLG substrate in a sequential three-stage evaporation process using Ga-In-Se, Cu-Se, and Ga-in-Se [24]. For the first sample (B01), the Se flux was maintained at 22 Å/s during CIGS growth, but this was changed to a 5 Å/s flux for the second sample (B02). The Se flux during three-stage coevaporation tended to influence the preferred orientation and morphology of the CIGS films. The B03 sample was prepared under experimental conditions similar overall to those of the first sample, but the 3^rd^ stage and finishing step were performed at slightly lower temperature (540°C) instead of 570°C. It is considered to influence the fabrication of the CIGS absorber layer. Different processing temperatures are often attributed to the variation of device cell efficiency. For the B04 sample, the SiO_*x*_ film was deposited by plasma-enhanced chemical vapor deposition (PECVD) at 200°C on an SLG substrate to interrupt the diffusion of Na elements from the substrate. The process condition of B04 was correlated to the low content of alkali metals in the CIGS absorber. Instead of a porous bottom Mo layer (100 nm), a Mo : Na film (5 wt.%) was used as the first bottom Mo film grown on the SiO_*x*_ film. The CdS buffer layer was deposited by chemical bath deposition for 10 min at 75°C. The intrinsic ZnO layer and an Al-doped ZnO layer, with thicknesses of 50 nm and 250 nm, respectively, were made by radio frequency sputtering. The configurations and conditions of deposition of the CIGS solar cells are summarized in Table 1.

### 2.2. Instrumental Analysis

ICP-AES and XRF were used to determine the bulk elemental composition of the CIGS absorber layers. ICP-AES measurements were obtained using an Agilent 720-OES emission spectrometer (Agilent Technologies, Santa Clara, CA, USA). Calibration of the ICP-AES was performed using an aqueous solution of 5% nitric acid and 1 ppm, 5 ppm, and 10 ppm solutions of Cu, In, Ga, and Se, respectively. For ICP-AES measurement, a 0.5 × 0.5 cm^2^ CIGS film was digested with HNO_3_ until the total dissolution of the CIGS films and the remaining Mo/glass substrate was removed from the solution. XRF measurements were completed using a ZSX Primus II (Rigaku, Tokyo, Japan), which was initially calibrated by a fundamental parameter (FP) based on the ratio of the four elements of the CIGS layer. For XRF measurement, CIGS standard was used and the entire 2 *μ*m thick CIGS films were sampled. A femtosecond laser ablation instrument (J200, Applied Spectra, USA) was coupled to an ICP-MS (iCAP Q, Thermo, USA) using the following conditions: a fluence of pulsed laser at 5.1 J/cm^2^ with a repetition time of 200 Hz and a 5 *μ*m diameter spot size. Each sample was scanned by the laser beam in a zigzag pattern over an area of 200 × 200 *μ*m^2^. At this condition, the complete ablation of a rectangular crater took about 3.85 s. The crater depth was more than 6 *μ*m so that the Mo layer and part of the SLG substrate and CIGS film were sampled. The same *fs*-LA-ICP-MS measurement was repeated over a 3 × 3 crater array for each CIGS film, and the average from the nine rectangular craters were then used to represent the ICP-MS isotope signal of each CIGS sample.

XPS measurement was performed with a PHI-5000 VersaProbe instrument (ULVAC-PHI, Japan) using Al K*α* (1486.6 eV) radiation. The Na 1s spectra were detected at a low pass energy of 58.70 eV. The depth profiles of Cu, In, Ga, and Se in the CIGS absorber layers were obtained using two different surface analytical instruments. The magnetic sector SIMS was completed using an IMS-4fE7 instrument (CAMECA, France). The CIGS samples were measured under an analyzed current of 20 nA with a raster size of 200 × 200 *μ*m^2^ and a mass resolution of 1800, applying 10 kV to a Cs^+^ primary ion gun and 4.5 kV to the sample to obtain an impact energy of 5.5 keV. AES data were obtained on a ULVAC-PHI 700 Scanning Auger microscope (ULVAC-PHI, Japan) under 5 kV at a current of 10 nA. AES intensities were acquired with the differentiated LMM transition at 920 eV, MNN transition at 405 eV, LMM transition at 1070 eV, and LMM transition at 1311 eV, for Cu, In, Ga, and Se, respectively. The AES depth profile was performed using a 3 kV Ar^+^ ion beam under a sputter rate of 52.5 nm/min for the SiO_2_ layer.

## 3. Results and Discussion

### 3.1. Composition of the CIGS Absorber Layers

The scanning electron microscopy (SEM) images of the four CIGS solar cells in cross section are shown in Table 1. The microstructures of all of the CIGS were faceted grains typical of absorbers grown in a three-stage process. The average thickness indicated in the table was calculated from several measurements collected on the same sample. Quantification of the bulk elemental concentrations of Cu, In, Ga, and Se and the depth distribution of these elements are essential to evaluate the accurate performances of the solar cells, which are related to solar-cell efficiency. The average concentrations of the four CIGS absorber layers were obtained by ICP-AES and XRF (Table 2). Differences between the atomic concentrations measured by ICP-AES and XRF were relatively low (within 1.4 at.%). The small difference between the results confirms the reliability of these analysis techniques to determine the bulk element compositions of CIGS absorber layers. Moreover, the measurement deviation can be mainly attributed to the analysis position and calibration method of each technique.

**Figure 1:**
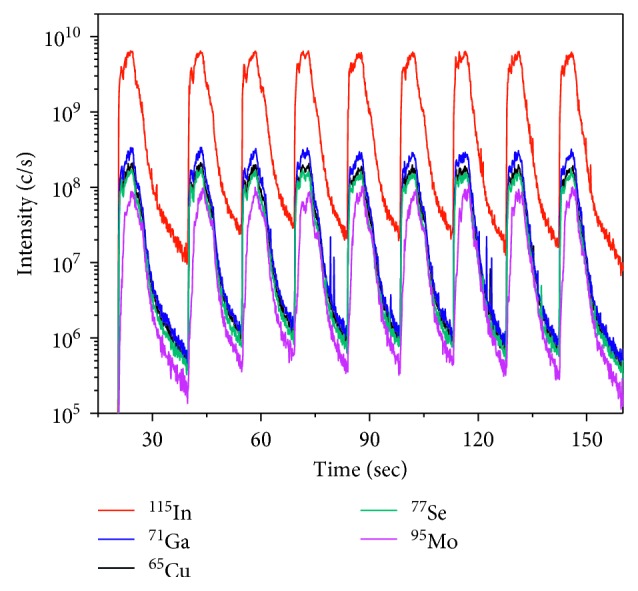
*fs*-LA-ICP-MS analysis of B04. Measurements were repeated nine times per sample, and the average value of the integral intensities was used (laser-repetition rate: 200 Hz, energy per pulse: 5.1 J/cm^2^, and crater diameter: 5 *μ*m).

**Figure 2:**
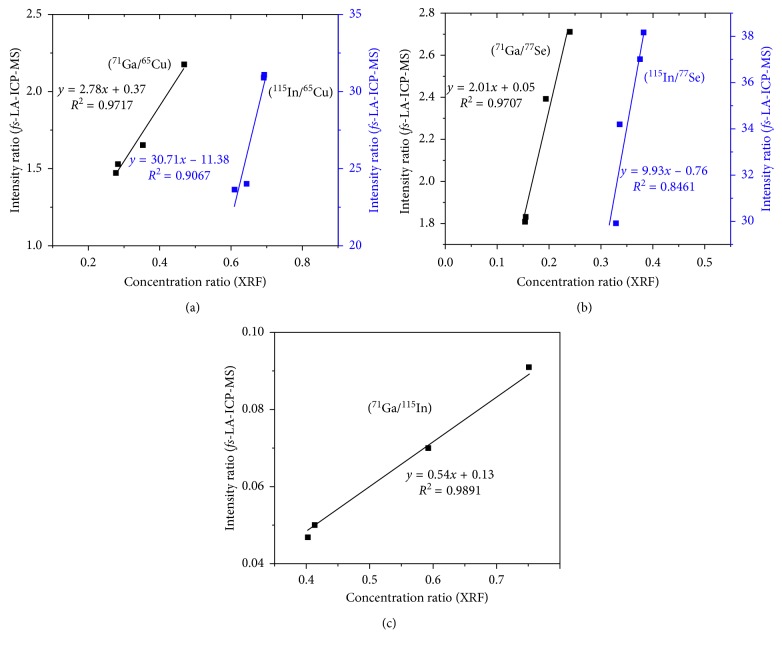
Calibration curves between the intensity ratios for *fs*-LA-ICP-MS and the concentration ratios for XRF measurement (^71^Ga/^77^Se, ^115^In/^77^Se, ^71^Ga/^65^Cu, ^115^In/^65^Cu, and ^71^Ga/^115^In ratios, respectively).

To find a fast, accurate, and reliable technique that can be readily applied for the quantification of CIGS absorber layers, *fs*-LA-ICP-MS was used and compared with other techniques. Calibration curves were generated for the *fs*-LA-ICP-MS signal ratios of the major elements with reference materials that were quantified by XRF [18]. Then, *fs*-LA-ICP-MS was performed to obtain the elemental concentration ratios of the samples. An *fs* laser was used to ablate the sample surface and to attain sequential access to the chemical composition. The *fs*-LA-ICP-MS measurements were performed for four different CIGS absorber layers, and the average values from the nine craters were used to represent the ICP-MS isotope signal of each absorber sample. The typical ICP-MS signals of ^115^In, ^71^Ga, ^65^Cu, ^77^Se, and ^95^Mo isotopes for B04 are shown in Figure 1. These signals indicated the four major elements of the CIGS absorber layers from the nine different craters. The repetitive responses of the ICP-MS results were consistent over the nine craters, indicating that the CIGS absorber layers had a fairly uniform compositional distribution. The relative standard deviation (RSD) of the intensities of each isotope signal was measured in the range of 3.7–15.8%. The *fs*-LA-ICP-MS technique provided reproducible quantitative results for the CIGS layers. The signal of ^95^Mo was also observed because the penetration depth of laser was deep enough to ablate both the CIGS layer and Mo layer under these experimental conditions. The intensities of each isotope signal are generated by the ablated amount of an element from the sample, which is related to the elemental concentration and the thickness of the CIGS layer. To improve the quantification of *fs*-LA-ICP-MS, the isotopic intensity ratios were calibrated with the relative concentration of the major elements in the CIGS layer. In Figure 2, the calibration curves for Ga/Cu, In/Cu, Ga/Se, In/Se, and Ga/In from the intensity ratios of the *fs*-LA-ICP-MS analysis are obtained and compared with the XRF elemental concentration ratios. The figure exhibits a good linear correlation between two of these methods. The *R*^2^ values of the linear fitting on the calibration curve for Ga/Cu and Ga/Se were close to 0.97, whereas the In/Se ratio had a lower value (0.85). Lower *R*^2^ values in the calibration curve, including for In, were also observed in the previous study by Lee et al. [18]. This result might be due to the mass difference and physical properties of In compared to other major elements (Cu, Ga, and Se).  Table 3 lists the average and RSD values of nine repeated measurements for elemental intensity ratios in *fs*-LA-ICP-MS. By using the intensity ratios instead of the absolute intensities, the RSD values of the nine measurements decreased to below 2% in average (maximum 2.87% for Ga/Se of B01 sample). These results imply that highly reproducible measurement can be performed with the elemental intensity ratios of *fs*-LA-ICP-MS at the optimal sampling condition. Therefore, the *fs*-LA-ICP-MS technique can be used to obtain accurate and rapid quantitative results for CIGS layer analysis.

### 3.2. Depth Profiling of the CIGS Absorber Layers

ICP-AES, XRF, and *fs*-LA-ICP-MS collect accurate and sensitive measurements, but these techniques cannot examine the depth distribution of elements. Thus, techniques such as SIMS and AES were used to investigate the elemental composition in a depth profile. In particular, SIMS provides high depth resolution and high mass resolution. This technique also has the advantage of high sensitivity in identifying trace elements such as sodium (Na) and potassium (K) that are closely related to cell efficiency.

**Figure 3:**
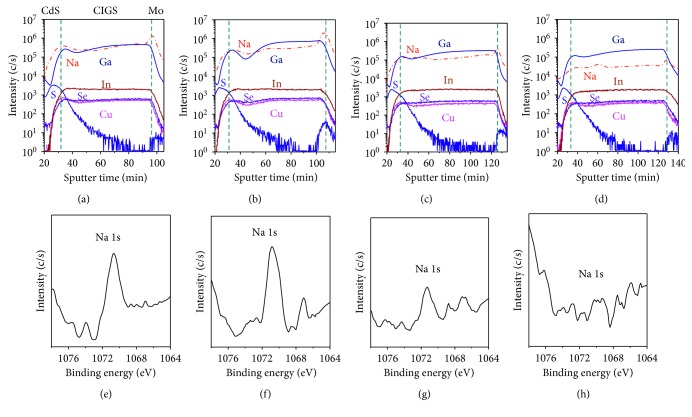
(a–d) SIMS depth profiles and (e–h) Na 1s peaks in the XPS spectra of the CIGS solar cells: (a, e) B01, (b, f) B02, (c, g) B03, and (d, h) B04.

Figure 3 shows SIMS depth profiles of characteristic ions in the CIGS solar cells. Dashed lines identify the layer position in the CdS/CIGS/Mo structure. The Cu and Se intensities were fairly uniform throughout the sputter time. However, severe Ga grading was observed, especially for the B01 and B02 CIGS layers. The distribution of Na in the SIMS depth profiling was obtained at high intensity and is clearly distinguishable due to the low detection limit of SIMS for trace elements. In contrast with B01, B02, and B03, the Na secondary ion intensity in the SIMS depth profile appeared lower in the CIGS layer for B04. To confirm the Na concentration on the CIGS surface, the as-prepared sample of CIGS/Mo on the SLG substrate was placed in the XPS system. As shown in Figures 3(e)–3(h), the CIGS thin films had Na peaks, with the exception of B04, which had a SiO_2_ barrier layer. No Na peak was observed in the XPS spectrum of B04 even though a Mo : Na layer was present on the SiO_2_ film. The decreasing Na intensity in B04 was mainly due to the presence of a SiO_*x*_ barrier layer on the SLG substrate, which limited Na diffusion during thermal treatment. The photovoltaic conversion efficiencies of B01 and B02, 14.6% and 14.3%, respectively, were greater than that of B04, measured at 10.3%. The Na distribution correlated with the change in conversion efficiency, which highlights the influence of alkali elements increasing the open-circuit voltage (*V*_OC_) [25, 26].

**Figure 4:**
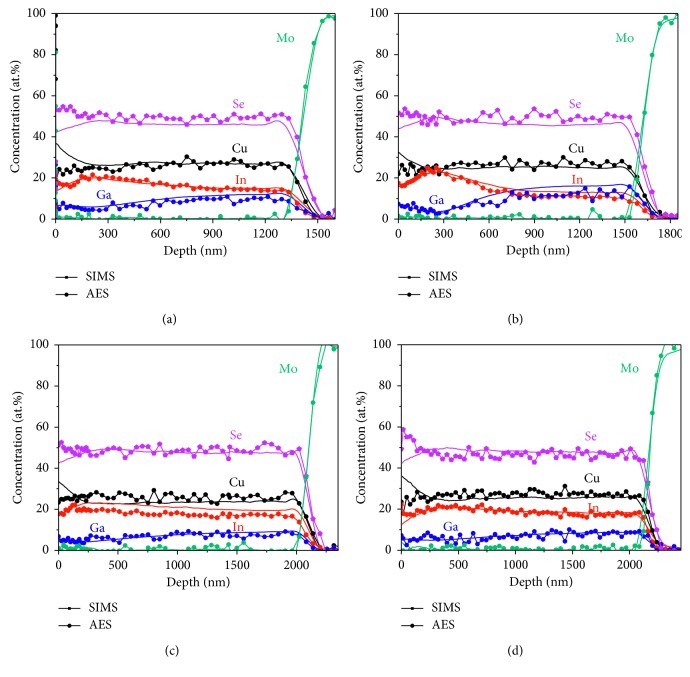
Overlaid compositional depth profiles of the CIGS absorber layers from SIMS and AES: (a) B01, (b) B02, (c) B03, and (d) B04.

All CIGS layers were quantitatively calculated using the ICP-AES concentration of B04 as a reference sample. The AES depth profiles were also quantitatively obtained in a way similar to the SIMS analysis of the CIGS layers. In Figure 4, the SIMS and AES results are overlaid to compare the compositional element distribution as a function of depth. The element distributions of SIMS and AES showed a good correspondence, except for the surface layer, which is the beginning of the profile in Figure 4. Large deviations in the interface layer appeared due to the surface effect, which is a classical artifact of the SIMS technique. The deviations can be reduced by detecting MCs^+^ cluster ions as shown in a previous study [24].

All of the calculated elemental compositions using SIMS and AES are reported in Table 2. The values in parentheses indicate the deviations of the average concentrations from AES and SIMS with the measured values from ICP-AES. The deviations from AES are relatively low except for the Ga concentration, especially for B02. The quantification errors might be caused by the analysis position of the sample, which is not uniform at the macrolevel, differences in the chemical compositions of the reference and analytical samples, or differences in the calibration methods of the specific analysis techniques.

### 3.3. Determination of Band Gap of the CIGS Absorber Layers

**Figure 5:**
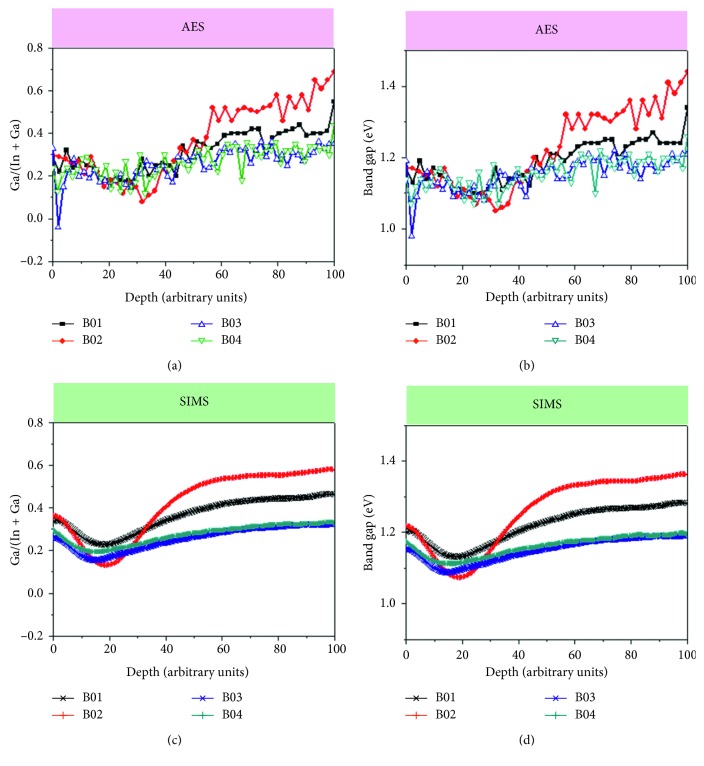
Determined band gap of the CIGS absorber layers from Ga/(In + Ga): (a) atomic ratios of Ga/(In + Ga) from AES; (b) band gap profiles calculated from AES; (c) atomic ratios of Ga/(In + Ga) from SIMS; (d) band gap profiles calculated from SIMS. Sputter time for each profile has been normalized to 100; actual sputter times varied from 65 to 95 min.

The depth band gap variation in the CIGS thin film caused by changes in the Ga/(In + Ga) ratio is commonly referred to as Ga grading [27–29]. The AES and SIMS composition measurements were used to calculate the gallium ratios, Ga/(In + Ga), and band gap profiles, as shown in Figures 5(a) and 5(c) and Figures 5(b) and 5(d), respectively. The band gap gradients in the samples reflected the gradings in the gallium ratios. B01 and B02 showed a notch structure in their band gap profiles, and their solar cells showed noticeable double-Ga grading, as expected in high-efficiency solar cells. The B01 and B02 solar cells showed similar device performance although B02 had a steeper Ga distribution profile (Figures 5(a) and 5(c)). Generally, the depth profiles of Ga grading tend to influence the cell efficiency of a CIGS solar cell: Ga-grading profiles are directly related to the distribution of the conduction band minimum. Basically, a double-graded Ga distribution (rising from the center toward both the front and the back surface) is known to enhance the cell efficiency because the bidirectional grading enhances the open-circuit voltage and the notch in the middle enhances the short-circuit current by lowering the optical band gap. However, if the Ga grading becomes severe (i.e., a very low Ga notch is formed), carriers are easily entrapped, which in turn increases carrier recombination and prevents carrier transport. Although reducing Se flux made the Ga grading steeper in B02, its device performance did not substantially changed from that of B01. These results implied that these device performances were determined by factors other than Ga grading itself (i.e., CIGS interface qualities). The gallium ratios, Ga/(In + Ga), in B03 and B04 were relatively flat, and these gallium distributions influenced their low cell efficiencies.

## 4. Conclusions

To understand the high-efficiency of Cu(In,Ga)Se_2_ solar cells, we accurately measured the compositions of Cu, In, Ga, and Se using ICP-AES and XRF, which have relatively low deviations within 1.4 at.%. The calibration curves were obtained by *fs*-LA-ICP-MS, which is a useful method for rapid elemental analysis and minimal sample consumption.

The depth profiles of the CdS/CIGS/Mo structure obtained through SIMS and XPS permitted us to highlight the minimal Na diffusion when a SiO_*x*_ layer was deposited between the SLG substrate and the Mo back contact layer. In contrast with B04, B01 and B02 were higher in photovoltaic efficiency, which is consistent with previous studies in which the alkali elements help to increase the open-circuit voltage.

The comparison of the SIMS depth profile with the AES depth profile confirmed the reliability of the depth analysis. Thanks to the accuracy of the depth distribution of the CIGS layers, the band gaps for four different CIGS absorber layers were determined using the Ga and In composition ratio along the film thickness. The B01 and B02 solar cells showed noticeable double-graded Ga distribution, which is known to enhance the cell efficiency by reducing the carrier recombination at the interface.

Comparative analyses of different CIGS absorber layers using various analytical techniques provide us with understanding of the accurate compositions, elemental distribution, and cell efficiency of CIGS solar cells. These results also give us the opportunity to select the proper analytical methods for the CIGS solar cells depending on the situations.

## Figures and Tables

**Table 1:** Configurations and conditions of the deposition processes for the CIGS samples.

Sample number		B01	B02	B03	B04
Configuration	n-TCO	i-ZnO/ZnO : Al	i-ZnO/ZnO : Al	i-ZnO/ZnO : Al	i-ZnO/ZnO : Al
	n-buffer	CdS	CdS	CdS	CdS
	p-absorber (*μ*m)	CIGS (1.70)	CIGS (1.98)	CIGS (2.24)	CIGS (2.21)
	Back contact	Mo	Mo	Mo	Mo/Mo : Na/SiO_*x*_

Condition of CIGS deposition (Å/s) (°C)		Normal Se flux (∼22) (570)	Low Se flux, (∼5) (570)	Normal Se flux (∼22) (540)	Normal Se flux (∼22) (570)

Cell efficiency (%)		14.6	14.3	11.7	10.3

SEM cross-section image		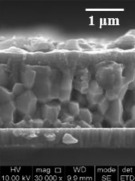	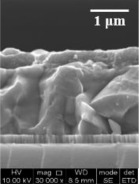	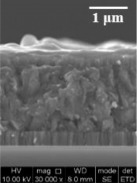	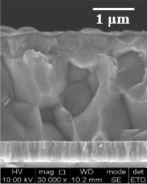

**Table 2:** Average elemental composition of CIGS absorber layers by ICP-AES, XRF, SIMS, and AES.

	Determination technique	Atomic concentration (at.%)							
		Cu		In		Ga		Se	
B01	ICP-AES	26.4	—	16.4	—	8.9	—	48.3	—
	XRF	26.5	(0.4)^a^	16.1	(−1.8)	9.3	(4.5)	48.1	(−0.4)
	SIMS	27.4^b^	(3.8)	16.6	(1.2)	9.6	(7.9)	46.5	(−3.7)
	AES	25.9	(−1.9)	16.2	(−1.2)	8.2	(−7.9)	49.7	(2.9)

B02	ICP-AES	25.2	—	15.6	—	11.7	—	47.5	—
	XRF	24.6	(−2.4)	15.8	(1.3)	11.5	(−1.7)	48.1	(1.3)
	SIMS	25.3	(0.4)	16.0	(2.6)	12.1	(3.4)	46.6	(−1.9)
	AES	26.2	(4.0)	15.1	(−3.2)	9.2	(−21.4)	49.5	(4.2)

B03	ICP-AES	26.5	—	19.2	—	7.4	—	46.9	—
	XRF	26.2	(−1.1)	18.1	(−5.7)	7.4	(0.0)	48.3	(3.0)
	SIMS	25.7	(−3.0)	20.0	(4.2)	7.0	(−5.4)	47.3	(0.9)
	AES	26.3	(−0.8)	17.9	(−6.8)	7.2	(−2.7)	48.6	(3.6)

B04	ICP-AES	26.2	—	18.9	—	7.1	—	47.7	—
	XRF	26.4	(0.8)	18.3	(−3.2)	7.3	(2.8)	48.0	(0.6)
	SIMS	26.2	—	18.9	—	7.1	—	47.7	—
	AES	26.2	—	18.9	—	7.1	—	47.7	—

^a^The numbers in parentheses represent the percentage deviation from the ICP-AES value. ^b^Calculated from the element peak intensities in the depth profile.

**Table 3:** Average and relative standard deviation (RSD) values of elemental intensity ratios using nine *f*s-LA-ICP-MS measurements.

Sample number	RSD (%)				
	Ga/Cu	In/Cu	Ga/Se	In/Se	Ga/In
B01	1.66^a^ (±1.70%)^b^	23.71 (±2.15%)	2.41 (±2.87%)	34.48 (±2.30%)	0.07 (±2.59%)
B02	2.18 (±2.70%)	24.08 (±1.13%)	2.71 (±2.18%)	29.94 (±1.34%)	0.09 (±2.69%)
B03	1.43 (±2.10%)	29.25 (±2.81%)	1.80 (±1.55%)	36.91 (±2.22%)	0.05 (±1.25%)
B04	1.48 (±1.16%)	31.12 (±1.18%)	1.81 (±0.93%)	38.22 (±1.30%)	0.05 (±1.13%)
Average of RSD	(±1.92%)	(±1.82%)	(±1.88%)	(±1.79%)	(±1.92%)

^a^The numbers represent the average of intensity ratios. ^b^The numbers in parentheses represent the RSD of intensity ratios.

## Data Availability

The data used to support the findings of this study are available from the corresponding author upon request.
